# Development of a Rigidity Tunable Flexible Joint Using Magneto-Rheological Compounds—Toward a Multijoint Manipulator for Laparoscopic Surgery

**DOI:** 10.3389/frobt.2020.00059

**Published:** 2020-04-28

**Authors:** Sousaku Kitano, Toshihiko Komatsuzaki, Ikuto Suzuki, Masamichi Nogawa, Hisashi Naito, Shinobu Tanaka

**Affiliations:** ^1^Graduate School of Natural Science and Technology, Kanazawa University, Kanazawa, Japan; ^2^Department of Clinical Engineering, Faculty of Health Sciences, Komatsu University, Komatsu, Japan

**Keywords:** medical robotics, manipulator, magnetic material, variable stiffness, electromagnetic device

## Abstract

Laparoscopic surgery is a representative operative method of minimally invasive surgery. However, most laparoscopic hand instruments consist of rigid and straight structures, which have serious limitations such as interference by the instruments and limited field of view of the endoscope. To improve the flexibility and dexterity of these instruments, we propose a new concept of a multijoint manipulator using a variable stiffness mechanism. The manipulator uses a magneto-rheological compound (MRC) whose rheological properties can be tuned by an external magnetic field. In this study, we changed the shape of the electromagnet and MRC to improve the performance of the variable stiffness joint we previously fabricated; further, we fabricated a prototype and performed basic evaluation of the joint using this prototype. The MRC was fabricated by mixing carbonyl iron particles and glycerol. The prototype single joint was assembled by combining MRC and electromagnets. The configuration of the joint indicates that it has a closed magnetic circuit. To examine the basic properties of the joint, we conducted preliminary experiments such as elastic modulus measurement and rigidity evaluation. We confirmed that the elastic modulus increased when a magnetic field was applied. The rigidity of the joint was also verified under bending conditions. Our results confirmed that the stiffness of the new joint changed significantly compared with the old joint depending on the presence or absence of a magnetic field, and the performance of the new joint also improved.

## Introduction

Laparoscopic surgery is rapidly replacing traditional open surgery because it is less painful, has shorter recovery times, and has better cosmetic results (Li et al., 2014). However, the current surgical instruments including graspers (Hannaford et al., 2013), laparoscopes, and trocars have disadvantages such as limited flexibility because of their rigid and straight structure. These mechanical restrictions cause internal interferences with other instruments and a limited view of the surgical area. To solve these structural problems, various types of surgical instruments and robots have been developed.

Among the developed methods, multijoint manipulators can improve the degree of freedom (DOF) of movement in a constrained space and thus are widely used not only in the medical field but also in search and rescue operations (Singh and Krishna, 2014). Surgical manipulators comprising rigid links and tendon-driven mechanisms are typical structures of flexible surgical devices (Kim et al., 2014; Rosen et al., 2017; Julie et al., 2019). However, in such types of manipulators, it is difficult to stiffen the arbitrary part of the robot while keeping the distal and the proximal ends floppy (Cianchetti et al., 2014). Takanobu et al. (2007) reported a multiple DOF manipulator for creating space inside the brain in minimally invasive surgery. The manipulator consists of discrete segments that can be tuned with respect to each other using wires and servo motors. However, the manipulator is very complex and expensive because it needs a considerably large automatic system and controller.

To overcome these drawbacks, we devised a new concept for a variable stiffness surgical manipulator using a magneto-rheological compound (MRC) (Tanaka et al., 2010). MRC is a smart material whose rheological properties can be quickly and reversibly tuned by applied magnetic fields. Typical MRCs are prepared by dispersing magnetic particles in a non-magnetic medium (de Vicente et al., 2011). The tunable property of an MRC results from the polarization induced in the suspended particles by an external magnetic field. Polarization causes particles to form columnar structures parallel to the magnetic field. Then, these chain-like structures become resistant to external forces (Carlson and Jolly, 2000). This unique feature makes MRCs useful in some applications such as brakes (Yu et al., 2016), dynamic vibration absorbers (Komatsuzaki et al., 2016), and even industrial robot grippers (Pettrtsson et al., 2010; Nishida et al., 2016). Current studies have mainly focused on dynamic properties of the material (Xu et al., 2013). In our variable stiffness manipulator, however, an MRC was employed under a compressive and static load. In our previous study (Tanaka et al., 2010), we fabricated a prototype of a single joint using silicon rubber-based magneto-rheological elastomer (MRE) and carried out experiments to verify the basic concept. We found that the stiffness of the joint can be changed by an external magnetic field. However, the rigidity difference of the joint under magnetic and non-magnetic fields was not satisfactorily high. The main reason for this is the dispersion state of the magnetic particles. In the MRE, silicon elastomer was used as the dispersion medium; therefore, the mobility of the magnetic particles was not as high as that of the fluid or gel. In this study, therefore, we changed the medium from the elastomer to a viscous fluid and prepared a novel magneto-rheological gel (MRG), expecting particles with a higher mobility and a consequent improvement in the rigidity difference of the joint. In addition, we evaluated the appropriate shape of the electromagnet through which a large magnetic field can be applied tothe MRG.

## Method

### Basic Concept of the Manipulator

**Figure 1:**
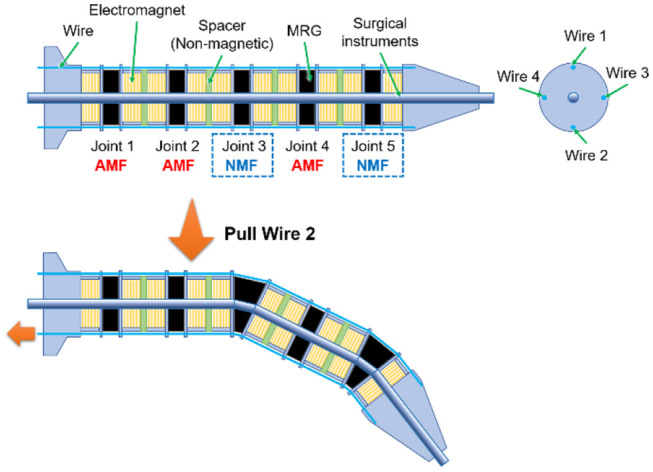
Structural outline of the variable stiffness manipulator using MRG.

The structural outline of the variable stiffness manipulator is shown in the upper part of Figure 1. It consists of MRG rings, electromagnets for generating the magnetic field, four wires, and spacers. As shown in the figure, the MRG rings are inserted between two electromagnets. Non-magnetic spacers prevent the magnetic field from leaking to the next joint. When an MRG ring is exposed to an attractive magnetic field (AMF), the stiffness of the joint increases. In contrast, when the applied current to the electromagnet is zero, it is exposed to a non-magnetic field (NMF), and the stiffness decreases. As a result, only the joint with NMF (e.g., joints 3 and 5) will bend when one of the four wires is pulled, as shown in the lower part of Figure 1.

### MRG Fabrication

**Figure 2:**
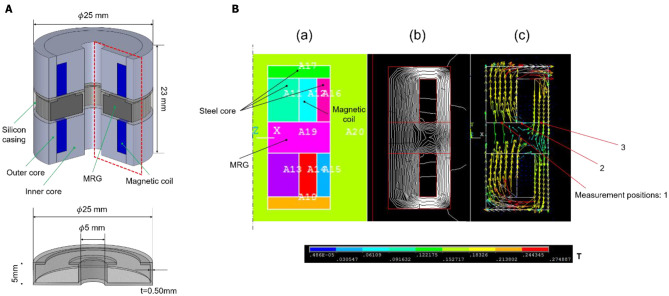
**(A)** Schematic of the prototype joint and silicon casing. **(B)** (a) Components of the simulation. (b) Simulation result of the line of magnetic force. (c) Simulation result of the magnetic flux distribution.

Carbonyl iron particles with an average diameter of 2 μm were selected as dispersed magnetic particles in this study because it is easily available and commonly used for general MRC. Commercial glycerol (Kenei Pharmaceutical Co., Ltd.) was used for the non-magnetic medium because it is non-volatile and has low reactivity with silicon, which is the material of the casing. MRG was fabricated with 83% weight fraction of carbonyl iron particles by direct mixing. The MRC was sealed in a flexible silicon casing (25 mm external diameter, 5 mm internal diameter, 5 mm height, 0.50 mm thickness, in Figure 2A) as it exhibits fluid property with NMF.

### Magnetic Field Analysis

The closed magnetic circuit prevents leakage flux and hence can generate an efficient magnetic field. Therefore, we designed a prototype joint using the closed magnetic circuit. A schematic diagram of the joint is shown in the left part of Figure 2A. The prototype joint comprises an MRG ring, inner and outer cores, and magnetic coils. The magnetic field was analyzed using finite element analysis software ANSYS. The components of the simulation model are shown in the left part of Figure 2B. The MRG ring and cores formed a closed magnetic loop for generating the magnetic field as shown in the middle part of Figure 2B.

### Elastic Modulus Measurement

To investigate the validity of the basic concept, we fabricated a prototype single joint based on the above magnetic field analysis. The inner and outer cores were made of pure iron. Polyurethane copper wire with a dimeter of 0.30 mm was used as the magnetic coil (186 turns, 20.6 mm external diameter, 15 mm internal diameter, and 7 mm height). The magnetic flux density at the measurement points shown in the right part of Figure 2B was measured using a tesla meter in the manufactured joint. When an electric current of 1.5 A was applied to the coil, the magnetic flux densities were 98, 86, and 73 mT for point 1, point 2, and point 3, respectively.

**Figure 3:**
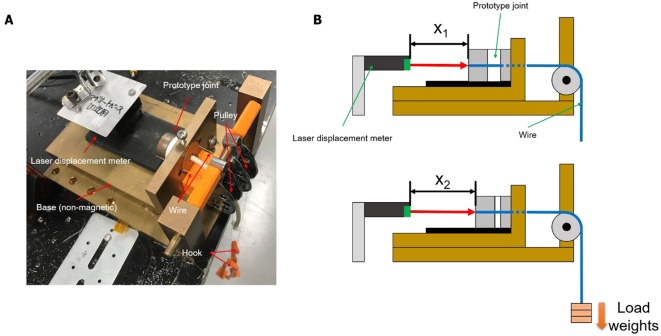
**(A)** Photograph of apparatus for loading weight and **(B)** compression test process.

The elastic modulus of the prototype joint was measured using the static compression test described below. Figure 3A shows the apparatus specially designed for loading weights on the joint. Figure 3B shows the compression test process. First, when the load on the left and right wires is 0 N as shown in the upper part of Figure 3B, the displacement of the joint in the compression direction is 0 mm, and the distance from the laser displacement meter (LB-02, LB-62, and KEYENCE) to the joint end face (x_1_) is measured. Then, a static compression load parallel to the axis of the joint is applied by adding the same number of weights to the left and right wires, as shown in the lower part of Figure 3B. Under this condition, the distance from the laser displacement meter to the joint (x_2_) is measured. The difference between x_1_ and x_2_ is the displacement of MRG due to the compression load, and the strain can be obtained by dividing this value by the initial length of MRG. As a result, we obtained the stress–strain relationships of the joint under different magnetic field conditions. The stress was calculated by dividing the load weight by the cross section of MRG.

### Flexion Angle Measurement

To ascertain whether the flexion angle of the joint can be tuned by varying the magnetic field, we measured the flexion angle of the joint under the following conditions. First, the weights (1.74 + 5 ×0.42 = 3.82 N) were loaded only to the right wire under AMF conditions, and the joint angle (θ) was manually measured from the photograph taken from the top. Then, the same procedures were carried out under NMF conditions. To calculate the angle from the photograph, we used ImageJ image processing software.

## Results

**Figure 4:**
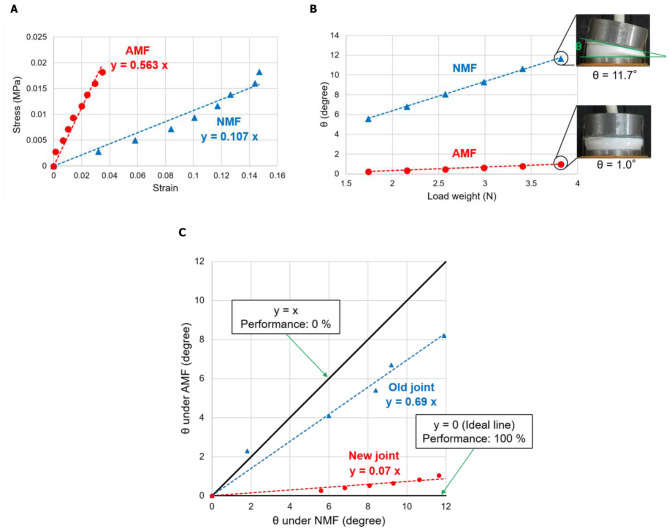
**(A)** Stress–strain relationships of the prototype joint under NMF and AMF, **(B)** relationships between load weight and joint angle (θ), and **(C)** relationships between θ under NMF and AMF.

The stress–strain relationships of the prototype joint under NMF and AMF are shown in Figure 4A. The figure shows that under NMF and AMF conditions, the stress was nearly proportional to the entire strain range. The slope of the line corresponds to the elastic modulus. The elastic moduli were determined to be 107 and 563 kPa under NMF and AMF conditions, respectively. MRG exhibits fluidity and easily deforms under load, particularly under NMF. Therefore, the value of the elastic modulus includes the effect of the elasticity of the silicon casing used in enclosing the MRG.

Figure 4B shows the relationships between the load weight to the right wire and the prototype joint angle (θ). In either case, θ increased with the increase in the load weight. As shown in the figure, the value of θ under AMF was considerably smaller than that under NMF, and the largest difference in θ was 10.6° at 3.82 N. Figure 4B also shows photographs of the prototype joint with different weights and magnetic field conditions. The photographs show that the stiffness of the prototype joint can be changed by applying a magnetic field; however, the maximum bending angle under NMF is small, and the value is considered to be insufficient for practical use.

## Discussion

By measuring the elastic modulus, we verified that the stiffness of the prototype joint using MRC can be tuned by an external magnetic field. The elastic modulus relatively changed, and the value under AMF was approximately 5.3 times larger than that under NMF. In our previous study, the elastic moduli of the joint under NMF and AMF were 108 and 187 kPa, respectively, and the relative change in the elastic modulus was 1.7 times (Tanaka et al., 2010). In the present study, the newly designed joint elastic modulus measured without applying a magnetic field is considered to be significantly affected by the silicon casing; however, a comparison between these two results confirmed that the stiffness of the joint was improved in the newly designed joint under AMF. The main factor responsible for this improvement is the change in the matrix of MRC from silicon rubber to glycerol. When the properties of MRC changes due to an external magnetic field, the chain-like structures of magnetic particles give rise to resistance to external forces (Carlson and Jolly, 2000). In the previous MRC, the movement of these particles would be hindered by the silicon rubber matrix. In contrast, because the glycerol used as the matrix is a liquid, it hardly prevents movement. Thus, particles could easily move along the magnetic lines, and the stiffness increased under AMF. In the newly fabricated MRG, we observed the sedimentation of particles caused by a density difference between the dispersed particles and the matrix. The stiffness of MRG increased as the particles inside are aligned in a chain with the application of a magnetic field. Therefore, if the particles settle in the casing, the distribution of the particles will have significant bias, and will be divided into a region where sufficient rigidity is exhibited when a magnetic field is applied and a region where it is not. This problem can be solved by introducing the so called “magnetic bias structure” using permanent magnets. Detail of this concept will be described later.

Various mechanisms have been reported for tuning the stiffness of joints (Loeve et al., 2010). Maghooa et al. (2015) reported a soft manipulator with antagonistic actuation. The manipulator can overcome the limitation of DOF and can create a wide workspace by combining a tendon-drive and pneumatic-actuation. However, each joint requires four wires to control the joints of the manipulator. This control method increases the complexity of the system, like the multiple DOF manipulator (Takanobu et al., 2007). In contrast, the system in our manipulator can be actuated using four wires regardless of the number of joints. Although Maghooa et al. (2015) did not mention the responsiveness of the various stiffness joints in their report, it is considered that it will take some time to change the pressure inside the manipulator. In contrast, the characteristics of MRC change rapidly with an external magnetic field (Goncalves and Carlson, 2007). Therefore, the use of MRC for tuning variable stiffness joints is considered effective.

From results of the flexing joint experiment, we confirmed the possibility of tuning the bending angle by applying a magnetic field. The difference in the angle according to the magnetic field conditions is important to realize a variable stiffness joint. Figure 4C shows the relations between the angles of the prototype joint under NMF and AMF. Each plot is a measurement result of the bending angle under NMF and AMF at each load weight. In this figure, the line of y = x with a slope of 1 indicates 0% performance because the bending angle at each load weight is the same regardless of the state of the magnetic field. In contrast, the line of y = 0 (an ideal line) with a slope 0 indicates 100% performance because the angle at each load weight when a magnetic field is applying is 0°. Hence, we quantitatively evaluated the performance of the joint using the slope in this figure. We confirmed that the performance of the new joint was (1–0.07) ×100 = 93% and that of the previous joint (Tanaka et al., 2010) was (1–0.69) ×100 = 31%. This result shows that the bending stiffness of the newly designed prototype joint improved under a magnetic field. However, the maximum bending angle in the experiment was 11.7°, which does not satisfy the range of motion of a single joint for clinical applications. For example, the tip of forceps used in robot-assisted surgery typically bends at approximately 90°, at the least. Because our current maximum bending angle of a joint is approximately 10°, nine joints are required to achieve this 90° bending. If the number of joints is more, more components will be required, thereby increasing the complexity of the system; therefore, improving the maximum bending angle and increasing the drive range per joint to be important. To solve this problem, the shape of the joint should be optimized to improve its flexibility. Moreover, we observed a high temperature rise in the magnetic coil due to joule heating. This problem can also be solved by employing the concept mentioned above, that is introducing the “magnetic bias structure.” This structure has been frequently used to decrease power loss due to joule heating (Jinji and Jiancheng, 2011). If strong permanent magnets are used to maintain the rigidity of the joints, and electric magnets are used only for bending the joints, the duration of power supply to the electric magnets could be considerably shortened. This will be highly effective not only for solving the power loss problem, but also the “sedimentation” problem mentioned earlier. This is because the MRG rings are always exposed to the strong magnetic field formed by the permanent magnets if the magnetic bias structure is used, and sedimentation of the magnetic particles can be prevented. We plan to implement this improvement in future research.

## Conclusions

In this study, we focused on improving the performance of a variable stiffness joint using MRG. To increase the joint stiffness upon applying a magnetic field, an MRG was prepared by mixing glycerol as a dispersion medium with carbonyl iron particles. A prototype single joint was fabricated based on magnetic field analysis. Results of preliminary experiments confirmed that the stiffness of the joint changed significantly compared with the conventional joint depending on the presence or absence of a magnetic field. However, several limitations of the joint, such as the large size and low movable range, need to be resolved for future practical use.

## Data Availability Statement

The raw data supporting the conclusions of this article will be made available by the authors, without undue reservation, to any qualified researcher.

## Author Contributions

SK drafted the manuscript and carried out the tests. ST and TK conceived the study. IS fabricated the joint. MN and HN performed the analysis. ST supervised the study and revised the paper.

## Conflict of Interest

The authors declare that the research was conducted in the absence of any commercial or financial relationships that could be construed as a potential conflict of interest.
